# Modulation of Voltage-Gated Sodium Channels from Sensory Neurons by Isoeugenol

**DOI:** 10.3390/ijms26167734

**Published:** 2025-08-10

**Authors:** David Ghim, Jehan Dib, Luiz Moreira-Junior, Joao Carvalho-de-Souza

**Affiliations:** 1 Arizona College of Osteopathic Medicine, Midwestern University, Glendale, AZ 85308, USA; david.ghim@midwestern.edu (D.G.); jehan.dib@midwestern.edu (J.D.); 2 Department of Physiology, College of Graduate Studies, Midwestern University, Glendale, AZ 85308, USA; 3 Superior Institute of Biomedical Sciences, State University of Ceara, Fortaleza 60714, CE, Brazil; 4 Department of Physiology and Biophysics, Biomedical Sciences Institute, University of Sao Paulo, São Paulo 05508, SP, Brazil

**Keywords:** voltage-gated sodium channels (VGSC), phenylpropanoids, isoeugenol, sensory neurons, local anesthetic mechanism

## Abstract

Isoeugenol is a phenylpropanoid that is commonly found in essential oils and has been commonly used as a flavoring agent in the culinary field and an anesthetic in fish. Yet despite its similarity to well-known eugenol, there is a lack of data regarding how isoeugenol would directly modulate neuronal excitability to interfere with pain signaling. Here, we studied the effects of isoeugenol on voltage-activated Na^+^ currents (I_Na_) as a means of starting to close the gap regarding the inhibitory properties of isoeugenol on neuronal excitability. We used rat dorsal root ganglia neurons under whole cell voltage clamp for the isolation of I_Na._. We show that isoeugenol effectively inhibits I_Na_ fully, reversibly, and in a dose-dependent manner. Our detailed analysis also indicates the direct interaction of isoeugenol with voltage-gated Na^+^ channels (VGSC) is likely state-dependent, as the inhibitory activity is enhanced by membrane depolarization. This effect is beneficial for pain management, as the drug would act more effectively as neuronal activity is promoted by membrane depolarization. Our data indicates a direct inhibition of VGSC by isoeugenol might constitute the main mechanism whereby this phenylpropanoid produces analgesia. This study serves as a basis for future approaches to deeply investigate the therapeutic potential of this drug or its derivatives.

## 1. Introduction

Essential oils are volatile and aromatic liquids extracted from plants, herbs, and spices and are composed of a diverse array of low molecular weight organic molecules. Based on their chemical structure, these compounds are typically classified into four main groups: terpenes, terpenoids, phenylpropanoids, and various secondary metabolites resulting from plant degradation [1]. Overall, phenylpropanoids have become well recognized for their effects on neuronal ion channels and receptors. For example, phenylpropanoids derived from *Cannabis sativa*, including β-caryophyllene, linalool, α-humulene, and geraniol, were effective in relieving neuropathic and inflammatory pain in mouse models of chemotherapy induced peripheral neuropathy. Mechanistically, these effects were shown to be mediated through adenosine A2A receptors in the spinal cord [2].

**Figure 1:**
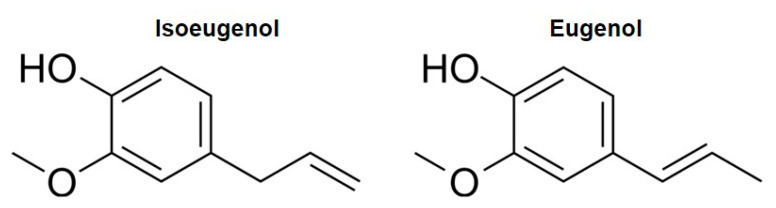
Chemical structures of isoeugenol and its position isomer eugenol.

Isoeugenol (IUPAC name 2-methoxy-4-propenylphenol, Figure 1) is a naturally occurring phenylpropanoid that is produced by plants as structural and defensive molecules. Isoeugenol is found in the essential oils from clove, ylang-ylang, jonquil, and nutmeg. The isoeugenol molecule can be modified to produce vanillin which has long served as a flavoring agent and is used in perfumes across industrial sectors [3,4]. One of its synthetic pathways in plants includes the reduction of a coniferyl alcohol ester [5]. Isoeugenol is a positional isomer of well-known eugenol that is used in dentistry as analgesic and antiseptic [6]. Isoeugenol and eugenol differ by the position of the unsaturation in the aliphatic chain of the molecule.

Regulatory bodies, including the U.S. FDA [7], recognize isoeugenol to be safe as a human and animal food additive [8]. In addition to the chemical versatility of isoeugenol supporting its widespread industrial application, its bioactive properties have also enabled incorporation into medical formulations. Isoeugenol has been shown to inhibit the growth of *Escherichia coli* and *Listeria innocua* with minimum inhibitory concentrations of 0.6 mg/mL and 1 mg/mL, respectively [9]. Moreover, isoeugenol has been demonstrated to possess cytotoxic activity against human cancer cell lines, including A549, DU-145, KB, and MCF7, suggesting mechanisms involving cell cycle arrest [10,11].

Isoeugenol is frequently used as a local anesthetic in fish due to its quick onset and effectiveness in inducing the loss of pain perception while not inducing stage IV anesthesia, even at higher concentrations, far above the therapeutic dose [12]. Studies with isoeugenol as a modulator of cell excitability are scarce; however, the results from other better-studied phenylpropanoids can be used to preliminarily predict the effects of isoeugenol on those tissues. Eugenol, the isoeugenol isomer, is likely one of the most well-studied phenylpropanoids. Regarding cell excitability only, eugenol has been shown to modulate voltage-gated Na^+^, K^+^, and Ca^2+^ channels [13,14,15,16]. Further, our group has recently demonstrated that eugenol acts on VGSC with similar affinity but with a different mechanism of action when compared with those of lidocaine [15]. Anethole is an isoeugenol analog with interesting biological activities. Anethole and isoeugenol possess the exact same aliphatic chain attached to the phenol ring, a prop-1-enyl, and other similar substituents in the phenol ring. Anethole has demonstrated multiple beneficial effects on humans, including anti-inflammatory, anticarcinogenic, and antidiabetic activities [17]. Anethole has been pointed out as a promising anticholinesterase agent that could be a potential lead in treating Alzheimer’s disease [18]. Recently our group has demonstrated the complex state-dependent modulation of VGSC by anethole [19]. Estragole, an anethole analog, also inhibits VGSC; however, it does so with less affinity [20,21].

Despite isoeugenol being widely used in various aspects of human society, including culinary applications to veterinary anesthesia, and its close structural resemblance to compounds like eugenol, the exact mechanisms through which isoeugenol interacts with neuronal pathways are not fully understood. Investigating how isoeugenol affects neural excitability through ion channels and its mechanisms of action may create new opportunities for therapeutic innovations. The chemical structure of isoeugenol shows potential as a foundational molecular scaffold for developing new neuromodulatory agents with enhanced effectiveness and selectivity. A better understanding of its pharmacodynamics has the potential to facilitate the design of improved treatments for pain, inflammation, and neurological disorders. The paucity in scientific knowledge about isoeugenol and neural tissue, especially the voltage-gated sodium channel, is the main motivation for this present work.

In this study, we investigated the modulatory effects of isoeugenol on neuronal excitability, specifically through its interaction with voltage-gated sodium channels (VGSC) from rat sensory neurons. Examining voltage-activated Na^+^ currents (I_Na_) from dorsal root ganglion neurons in the presence of isoeugenol, we found rich data on how this molecule may inhibit neuronal excitability. Isoeugenol inhibits I_Na_ reversibly modulating VGSC in a state dependent manner. Isoeugenol inhibits the resting state of the VGSC with an IC_50_ of about 1 mM. In addition, the inhibition is enhanced by depolarized conditions of resting potential (holding potential or a conditioning pre-pulse period in voltage clamp), likely by interacting with a different conformation of the VGSC that appears on those voltages. Our seminal data paves the way for advancing the knowledge about isoeugenol and cell excitability, and it suggests isoeugenol is a suitable molecular scaffold for drug development with the advantage of the existing knowledge attesting to its low toxicity in humans.

## 2. Results

### 2.1. Isoeugenol Modulates I_Na_ in a Dose-Dependent Manner

**Figure 2:**
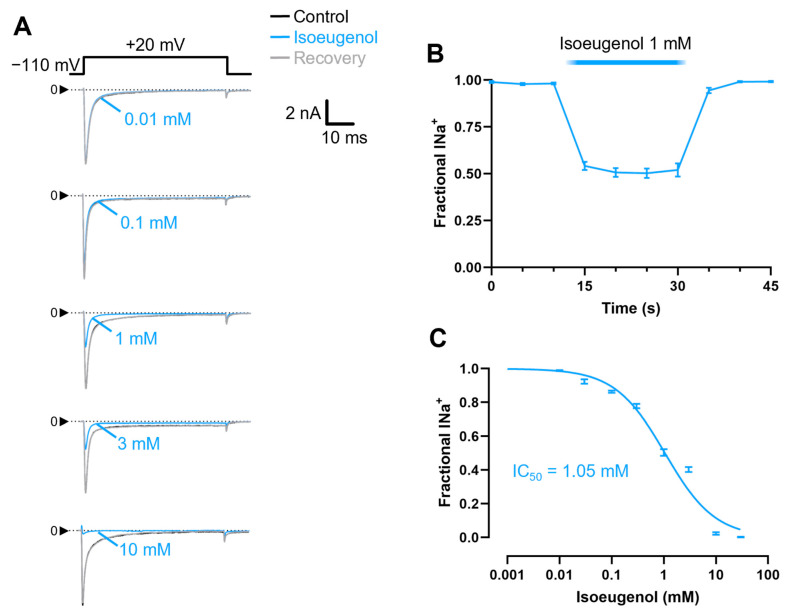
Isoeugenol inhibits voltage-activated sodium current (I_Na_) in a concentration-dependent manner. (**A**) Representative traces of I_Na_ recorded with the indicated voltage clamp protocol used at 0.2 Hz under control conditions (black), during application of different concentrations of isoeugenol as indicated (blue), and after drug washout (gray). (**B**) Time course of I_Na_ inhibition by isoeugenol 1 mM as indicated, followed by a recovery period after drug washout. These experiments were conducted with time series of membrane depolarizations as in (**A**), applied at every 5 s from holding potential. § One-way ANOVA, Dunnett’s multiple comparisons test vs. point at 10 s used as control (↓) for this test, *p* < 0.0001 (n = 8). (**C**) Dose response curve showing the fractional inhibition of I_Na_ across a range of isoeugenol concentrations. Data in the dose response curve is expressed as average (no symbols), and the bars indicate the SEM. For each dose–response concentration at least six different experiments (n > 6) were used. The continuous line is a plot of the Hill equation (Equation (1), see Section 4) that best represents the data. The IC_50_ is 1.05 mM, and the Hill slope is 0.9. The IC_50_ and correlation coefficient R^2^ are shown in the inset.

Isoeugenol inhibits I_Na_ promptly, fully reversibly, and in a dose-dependent manner (Figure 2). We used 0.2 Hz time series of membrane depolarizations to +20 mV from holding potential to test the effects of isoeugenol on the peak of I_Na_, to build a dose–response curve (Figure 2A). Fractional (normalized) I_Na_ levels (peaks) from individual experiments before and during addition of isoeugenol 1 mM to the experiment, as well as after drug removal, were plotted as a function of the time (Figure 2B) and as a function of the isoeugenol concentration for a dose–response relationship (Figure 2C). Upon addition to an experiment, isoeugenol inhibits I_Na_ in less than a 5 s interval. Similarly, isoeugenol inhibitory effect disappears in less than 5 s upon drug removal. The dose–response data was fitted with the Hill equation with a variable slope (Equation (1), see Section 4) for the IC_50,_ 1.05 mM, and the Hill slope, 0.9 (Figure 2C). These numbers will be picked up in the discussion session.

### 2.2. Isoeugenol Accelerates I_Na_ Activation

**Figure 3:**
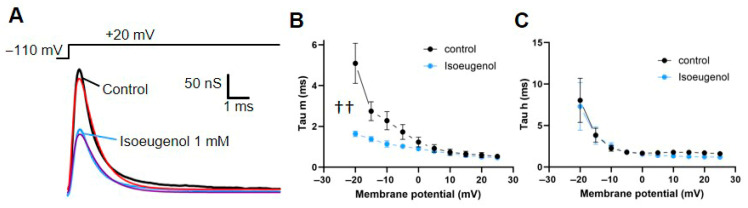
Isoeugenol effect on sodium conductance. (**A**) Representative sodium conductance traces recorded at +20 mV under control conditions (black) and with 1 mM isoeugenol (blue). The Hodgkin and Huxley model was fitted to the data (red and purple traces in the figure) using Equation (3) (see Section 4). (**B**,**C**) Voltage dependence of activation (Tau m) and inactivation (Tau h) time constants under control (black) and isoeugenol (blue) conditions pooled from six different experiments (n = 6). Plotted data is average and the vertical bars indicate SEM. †† Two-way ANOVA, significant variation in control vs. isoeugenol data, *p* < 0.0001, n = 7.

We converted the time course of activation and inactivation of I_Na_ into conductance (Ohm’s law, Equation (2)) and fitted the result (the time course of the Na^+^ conductance) with the Hodgkin and Huxley model (Equation (3)) (Figure 3A). We evaluated the I_Na_ activation time constant Tau m as well as inactivation time constants Tau h over several voltages spanning from −20 mV to +25 mV in the absence (control) and in the presence of isoeugenol 1 mM. Our data show that the activation time constant Tau m (Figure 3B) but not the inactivation time constant Tau h (Figure 3C) is significantly changed by isoeugenol. Tau m is accelerated in the presence of isoeugenol.

### 2.3. Isoeugenol Does Not Affect the Activation of I_Na_ by Voltage

**Figure 4:**
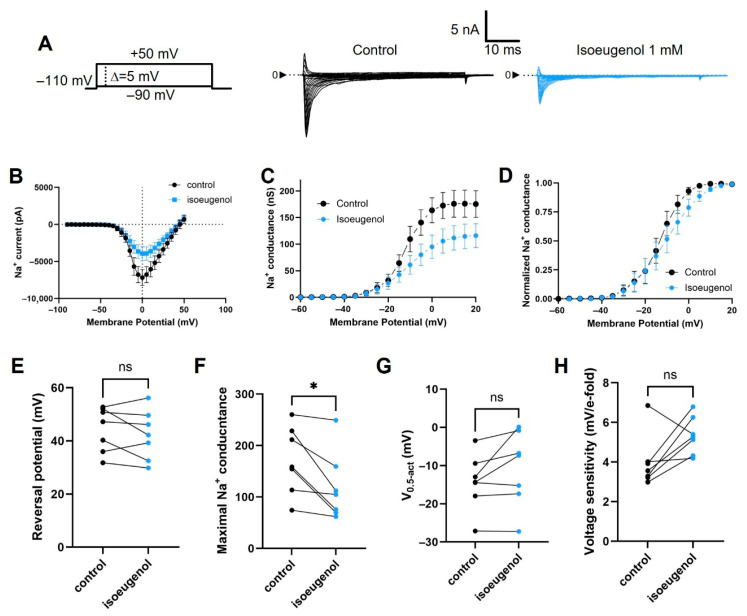
Effects of isoeugenol on the I_Na_ activation. (**A**) Representative family of I_Na_ recorded under voltage clamp using the indicated voltage protocol, in the absence (control) and in the presence of isoeugenol 1 mM. (**B**) I–V relationship of I_Na_ peaks. (**C**) For each cell analyzed in each condition, I_Na_ values were transformed in conductance, the grouped values averaged and plotted against the membrane potential for conductance–voltage relationships. (**D**) Normalized conductance–voltage relationships are shown to highlight the respective voltage dependences. (**E**–**H**) Paired summary data from several individual cells (n = 7) comparing biophysical parameters of the I_Na_ before (control) and after isoeugenol exposure: (**E**) reversal potential, (**F**) maximal sodium conductance, (**G**) half-activation voltage, and (**H**) voltage sensitivity. Paired *t* test with data from individual cells were performed (see text for details); ns: not significant, * *p* = 0.0183.

**Figure 5:**
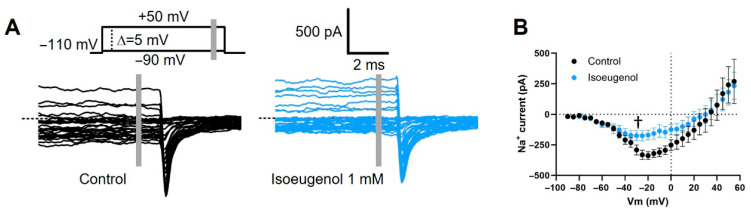
Isoeugenol inhibits persistent I_Na_. (**A**) Representative family of persistent I_Na_ after approximately 50 ms depolarization recorded under voltage clamp using the indicated voltage protocol, in the absence (control, black) and in the presence of isoeugenol 1 mM (blue). The data were collected from the position indicated by the vertical gray line. (**B**) Averaged I–V relationship from multiple cells (n = 7) showing persistent I_Na_. All plotted data indicate mean values, and the vertical bars are representative of SEM (n = 6). ^†^ Two-way ANOVA, significant variation control vs. isoeugenol, *p* < 0.004.

The modulatory effects of isoeugenol 1 mM on the current-to-voltage (I–V) relationship for I_Na_ were limited to the inhibition of maximal Na^+^ conductance (Figure 4). Current values used for the I–V relationship, acquired at 5 mV incremental/decremental steps ((Figure 4A and Figure 5A), were transformed into conductance by using Ohm’s law (Equation (2)). Conductance data from individual cells (control and in the presence of isoeugenol) were fitted with the Boltzmann formalism (Equation (4)). From the fitting method, we estimated the V_0.5-act_, which is the midpoint of the inactivation curve and the voltage sensitivity that is the maximal slope of the curve. Isoeugenol does not change the reversal potential of I_Na_, the voltage dependence of the current measured by the voltage that activates 50% of the maximal Na^+^ conductance (V_0.5-act_), or its activation voltage sensitivity measured as the minimal voltage change necessary to increase Na^+^ conductance by *e*-fold per our paired comparisons of individual cells data under control and in the presence of isoeugenol 1 mM (Figure 4E,G,H). Averaged fitting parameters from the above analysis are shown in Table 1 for reference.

Similarly to the inhibition of the peak I_Na_, isoeugenol blocks the persistent, non-inactivated I_Na_ after approximately 50 ms depolarization pulses (Figure 5). The inhibition of these persistent I_Na_ by isoeugenol is like the one produced by this drug on the peak I_Na_: about 50% using isoeugenol 1 mM.

### 2.4. Isoeugenol Remarkably Affects the Inactivation of I_Na_ by Voltage

**Figure 6:**
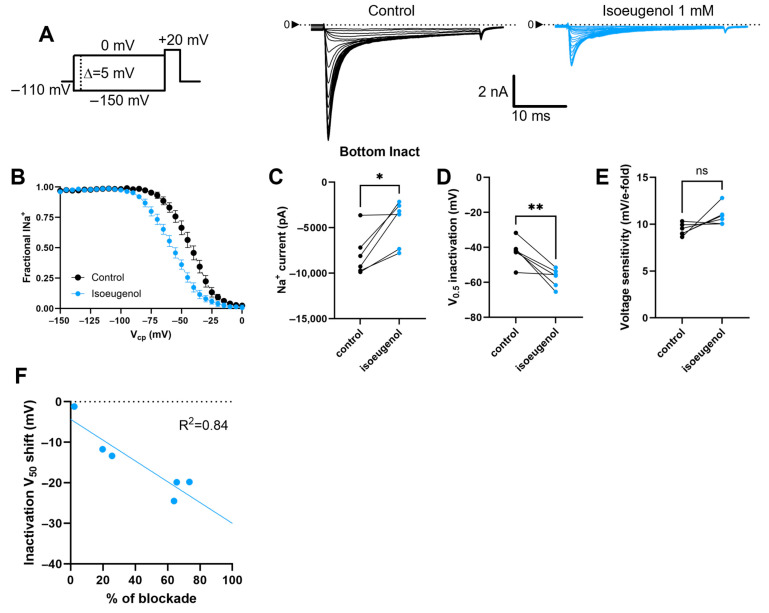
Isoeugenol effect on inactivation of I_Na_. (**A**) Representative current traces from voltage-clamp recordings at +20 mV, after a 100 ms conditioning period from −150 to 0 mV. I_Na_ are shown in control conditions (black) and with 1 mM isoeugenol (blue). (**B**) Steady-state inactivation curves showing a hyperpolarizing shift in V_0.5_ with isoeugenol. (**C**–**E**) Paired data from individual cells comparing (**C**) peak sodium current, (**D**) half-inactivation voltage, and (**E**) voltage sensitivity under control and isoeugenol conditions. Paired *t*-test; ns, not significant; * *p* < 0.0157; ** *p* < 0.0072 (n = 6). (**F**) Linear correlation between individual values of V_0.5-iNact_ shifts induced by isoeugenol 1 mM and the correspondent level of I_Na_ % of inhibition (Simple linear regression, slope different from zero, *p* = 0.002, n = 6).

We used a voltage clamp protocol consisting of a 100 ms conditioning period at different voltages, with 5 mV incremental/decremental steps (Figure 6A), for current inactivation, followed by a 30 ms period at +20 mV for the activation of the remaining current. We plotted the peaks of the remaining currents against the voltage during the conditioning period for the inactivation curves (Figure 6A,B). In addition to the expected inhibition of the currents’ peaks (Figure 6C), isoeugenol at 1 mM produced a negative shift in the inactivation curves as evidenced in Figure 6D without significant changes in the voltage sensitivity of the process. This voltage sensitivity is the minimal voltage change that enhances inactivation of the currents by e-fold (Figure 6E). I_Na_ data from individual cells (control and in the presence of isoeugenol) were fitted with the Boltzmann formalism (Equation (5)). From the fitting method we estimated the V_0.5-iNact_, which is the midpoint of the inactivation curve and the voltage sensitivity that is the maximal slope of the curve. Isoeugenol at 1 mM induces a shift in the midpoint of the inactivation curves (V_0.5-iNact_) by −15.06 ± 3.368 mV (Paired *t* test, *p* = 0.0066). The shift in the inactivation V_0.5-iNact_ induced by isoeugenol is linearly correlated with the intensity of the I_Na_ inhibition, displaying an R^2^ = 0.84 in our analysis (Figure 6F). I_Na_ voltage dependent inactivation parameters are shown in Table 1.

### 2.5. Isoeugenol Changes the Recovery from Inactivation of I_Na_

**Figure 7:**
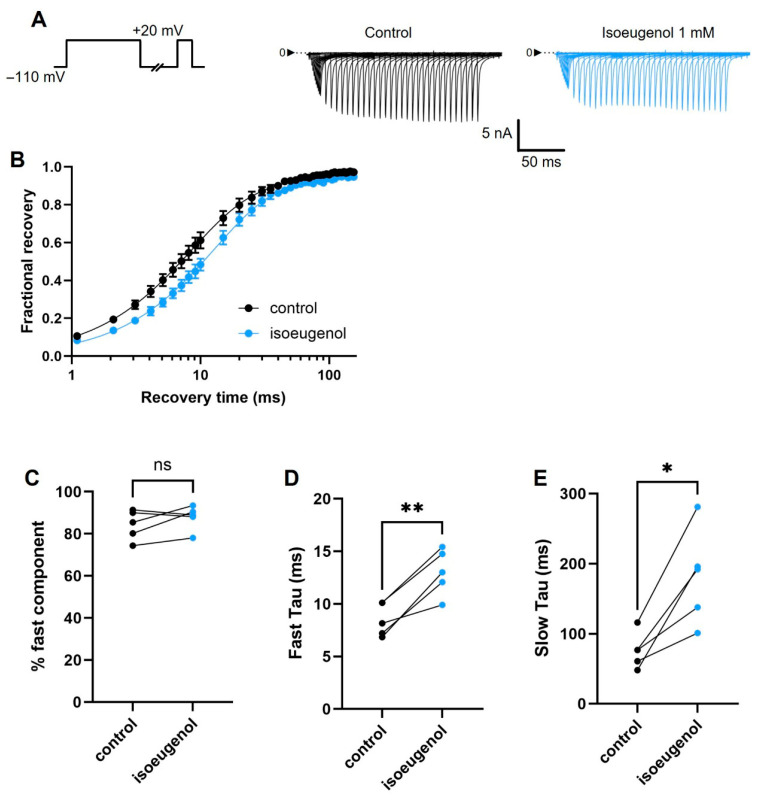
Effects of isoeugenol on the recovery from inactivation of I_Na_. (**A**) Representative traces of I_Na_ recovered from inactivation in control condition (black) and with isoeugenol 1 mM (blue). The voltage protocol consisted of an initial 50 ms + 20 mV pulse to inactivate I_Na_. Next, a varying period (1–150 ms) at holding potential gradually recovered I_Na_ from inactivation. Finally, another depolarization to +20 mV assessed the fraction of I_Na_ recovered from inactivation. (**B**) Fractional values of recovered I_Na_ from five independent experiments (n = 5) were averaged and plotted against the respective recovery period in control condition and with isoeugenol 1 mM. (**C**–**E**) Individual cells’ data were fitted with a double exponential (Equation (5), see Section 4) for a fast and a slow component during the recovery from inactivation process. Best fit data were individually plotted: (**C**) % fast component amplitude, (**D**) fast component time constant (Fast Tau), and (**E**) slow component time constant (Slow Tau). Paired *t*-test; ns, not significant; * *p* = 0.0119; ** *p* = 0.0037.

The rate of recovery from inactivation of I_Na_ is a parameter that is directly related to refractory periods in neurons. We evaluated the rate of recovery of inactivation of I_Na_ by using the classical three pulses voltage protocol. First, we applied a strong depolarization to +20 mV to fully inactivate I_Na_ in 50 ms. Next, at a varying period at a holding potential of −110 mV, the currents are allowed to recover from inactivation to be reprimed for re-activation, which we measure by a second depolarization to +20 mV at the end of every sweep of this time series. Isoeugenol modestly but significantly delays the recovery from the inactivation process in I_Na_ by changing both the fast and the slow processes (Figure 7A,B). Isoeugenol delays the fast process, delaying this process from a time constant of 8.5 ± 0.70 ms during control, to 13.0 ± 0.98 ms when the drug is present (Paired *t* test, *p* = 0.0119) (Figure 7D). The slow process of recovery from inactivation is delayed by isoeugenol at 1 mM from a time constant of 75.9 ± 11.40 ms during control, to 181.6 ± 30.51 ms in the presence of isoeugenol (Paired *t* test, *p* = 0.0037) (Figure 7E). Table 2 shows a summary of these results.

### 2.6. The Modulation of I_Na_ by Isoeugenol Is Not Intensified by High-Frequency Depolarizations

**Figure 8:**
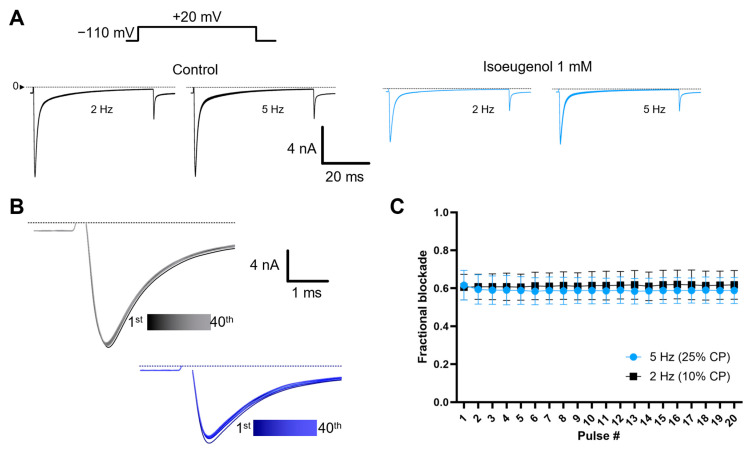
Inhibition of I_Na_ induced by isoeugenol is not use-dependent. (**A**) Representative I_Na_ traces recorded during 2 Hz and 5 Hz repetitive stimulation with 50 ms pulses to +20 mV from holding potential of −110 mV under control conditions (black) and in the presence of 1 mM isoeugenol (blue). No sign of frequency dependent accumulation of inhibition is apparent. (**B**) Zoomed traces from A in the presence of isoeugenol showing first through twentieth I_Na_ traces for 2 Hz stimulation and first through fortieth traces from 5 Hz stimulation. (**C**) Averaged peak I_Na_ data and SEM during stimulations at 2 Hz and at 5 Hz.

To evaluate if isoeugenol would have its modulatory effects on I_Na_ enhanced by high frequency of depolarizations, we used a simple protocol consisting of a series of 50 ms depolarizations to +20 mV, from a holding potential of −110 mV, at 2 Hz and 5 Hz. Common knowledge says that such frequency-dependent effect is associated with a higher affinity of a drug to the inactivated state of the VGSC compared to that of during the resting states of the channels. For these experiments we refrained from using the p/N protocol to avoid biases in the recovery from inactivation processes of I_Na_ (see Section 4). Neither 5-s stimulation at 2 Hz nor 5-s stimulation at 5 Hz were sufficient to significantly enhance the modulatory effects of isoeugenol. The peak current of the inhibited I_Na_ by isoeugenol at 1 mM was maintained throughout either series (Figure 8).

## 3. Discussion

Isoeugenol is one of the least studied plant-derived phenylpropenes regarding their effects on excitable cells. In human culture, isoeugenol is added to foods as a flavoring agent, and to household cleaning agents and hygiene products for its sweet, spicy, and floral fragrance properties [22]. It is important to note that there is no evidence of carcinogenic activity by isoeugenol in these comprehensive studies. Our novel data shown here unequivocally indicate that isoeugenol interferes with neuronal excitability by exerting an inhibitory effect of VGSC, the membrane proteins that mediate Na^+^ entry into cells to quickly depolarize their membrane potential for the upstroke phase of the action potential. Since all neural activities depend upon action potentials generation, isoeugenol has the potential to interfere with all functions of the nervous system and we reasoned this effect should be investigated.

A variety of phenylpropanoids like isoeugenol have been shown to be active on excitable cells from mammals, especially on neurons. Hence, it is imperative to study the effects of isoeugenol on excitable cells since overall this class of substances is well known to modulate ion channels [23,24]. To our knowledge, isoeugenol has never been studied as a modulator of VGSC despite its uses and its similarities with eugenol, a phenylpropene known to modulate these channels [14,15,25,26]. Isoeugenol is a position isomer of eugenol. In isoeugenol, the unsaturation is in carbon 1 of the aliphatic chain, while in eugenol the unsaturation is in carbon 2 of this moiety (Figure 1). According to our data, the position of the unsaturation in the aliphatic chain is associated with the affinity of these molecules to VGSC expressed by sensory DRG neurons. Isoeugenol inhibits VGSC with an IC_50_ of 1 mM (this study), while eugenol does so with an IC_50_ of 2 mM [26]. Similar affinity enhancement effect appears with another isomer pair of phenylpropanoids: anethole (1-Methoxy-4-(1-propenyl) benzene), recently studied by our group, and estragole (1-Methoxy-4-(2-propenyl)-benzene). These two molecules differ by the same shift in the position of the aliphatic chain unsaturation, from carbon 1 to carbon 2 [19,21]. Like in isoeugenol vs. eugenol analysis, anethole, the molecule with the same aliphatic chain as isoeugenol, inhibits I_Na_ with higher affinity than estragole that has the same aliphatic chain as eugenol. We hypothesize that the binding properties of these phenylpropanoids to VGSC are determined by the quality of the unsaturated aliphatic chain of the molecules.

DRG neurons express a variety of VGSC, preferentially the alpha subunits Nav1.7, Nav 1.8 and Nav1.9, but Nav1.1, Nav1.2 and Nav 1.6 are also present [27,28,29,30,31,32,33]. In neonatal rats, DRG neurons undergo significant changes in the expression of VGSC. Nav1.1 is absent during early embryonic development but emerges by the third post-natal day and increases slightly afterwards. Nav1.2 is consistently expressed at moderate levels from embryonic to the thirtieth postnatal day. Nav1.6 and Nav1.7 are both strongly expressed postnatally. Nav1.6 gradually increases across postnatal development, while Nav1.7 shows consistent expression from the seventeenth embryonic day and onward, particularly in larger DRG neurons. The expression of Nav1.8 and Nav1.9 begins during late embryonic development, on the fifteenth and seventeenth embryonic days, respectively, and becomes widespread across DRG neurons by the seventh post-natal day. Like Nav1.8, Nav1.9 is highly expressed in small-diameter, unmyelinated neurons that give rise to C-fibers and are involved in pain signaling. These channels play unique roles in shaping the DRG neuron function during development, and investigating them in their physiological environment rather than artificial expression systems is essential for studying how they operate and interact under native conditions [27,29,34,35,36,37]. Establishing specific binding affinities between isoeugenol and different VGSC alpha subunits and molecular complexes was not in the scope of the present study. Nevertheless, using natively expressed VGSC has its advantages for exploration studies since the channels are tested in their natural environment, the membrane of sensory neurons, and in the context of their supramolecular protein complexes. In other words, DRG neurons contain VGSC in their physiological environment.

A recent study using electrophysiological recordings from the Mauthner neuron, a motor neuron in goldfish, assessed the inhibitory properties of isoeugenol on neuronal excitability [12]. Isoeugenol is routinely used as an anesthetic in fish farming. The study found that a concentration of 10 mL/L (approximately 61 μM) was sufficient to inhibit the neuronal excitability of those neurons from goldfish. In vitro, the 61 μM of isoeugenol found by the study carried by Machnik and cols. is considerably lower than the effective concentrations of 1 mM we found to inhibit VGSC from mammals (rats). We reason these differences might be due to different aspects of our studies such as the types of experiments, VGSC tissue distribution, possible different ion channel subtypes, or VGSC drug sensitivity. Nevertheless, overall, these discrepancies likely reflect fundamental differences between our studies. Differences between recording action potentials from the neurons and their ionic currents certainly play a role. Action potentials firing can be inhibited by blocking only a fraction (e.g., 10%) of VGSC, whereas the affinity-related parameter IC_50_ from our voltage clamp data is associated with an inhibition of 50% of the channels. The exposure period is also important to explain these discrepancies. Our assessment is rapid (<30 s) and may be limited to a direct effect of isoeugenol on the VGSC in the membrane of the neurons. Differently, a several minutes experiment would allow for additional effects, e.g., intracellular effects on other targets, due to lipophilicity of the molecule that allows diffusion through the cell membrane. Importantly, our experiments showed no changes in the input resistance (as proxied by the reversal potential of our I–V curves) that would indicate the membrane electrical properties are modified by the drug being assessed. Finally, these discrepancies can also be explained based on fundamental differences in phylogeny and physiology, highlighting the importance of species-specific considerations when evaluating VGSC modulators like isoeugenol.

An additional interesting difference between the modulation of VGSC by isoeugenol and by eugenol is the slope of the dose–response curve. Numerically, this parameter is the Hill coefficient of the Hill equation when fitted to the data. Isoeugenol inhibits I_Na_ with a Hill coefficient of around 1, while eugenol does so with a Hill coefficient of 2 [26]. This suggests that the stoichiometry of the interaction between isoeugenol and VGSC could be 1:1, but this interpretation of the Hill coefficient is mostly applicable to orthosteric inhibitors such as pore blockers [38,39]. It remains unclear based on our data whether isoeugenol modulates VGSC by binding to multiple sites on the channels’ proteins. Factually, VGSC inhibition can take place when a molecule directly obstructs the conductance pore, when it prevents the voltage sensor of the channels from moving or even by decoupling the voltage sensors pore domain to prevent the activation of the ionic conductance of the channel. Nonetheless, it is interesting that a shift in the position of the unsaturation in the aliphatic part of these isomers’ molecules (isoeugenol vs. eugenol) is enough to produce such a remarkable change in its inhibitory pattern.

Further experiments on the inhibitory effects of isoeugenol on VGSC suggested a state-dependent interaction between isoeugenol and the channels’ molecules. Isoeugenol interacts with the resting state of the channels, existing at −110 mV, our holding potential, to pre-inhibit I_Na_ when the channels open in response to membrane depolarization (Figure 2, Figure 3, Figure 4 and Figure 5). In other words, with parsimony, we hypothesize isoeugenol simply prevents Na^+^ conductance without trapping the channels in a structurally closed channel. Our data indicates this inhibition takes place with an IC_50_ of 1 mM. However, when the membrane potential is depolarized for a brief period of 100 ms prior to the activating pulse to +20 mV, we observed isoeugenol seems to be more potent to inhibit I_Na_. These experiments were performed to build inactivation curves as shown in Figure 6. As demonstrated, a more intensive inhibition of I_Na_ by isoeugenol is reflected as a negative shift in the inactivation curve when this drug is present in the experiment. (Figure 6). Interestingly, isoeugenol becomes more effective at inhibiting I_Na_ when conditioning pre-pulses at voltages not related to open channels are applied, such as −90 mV and −40 mV. Overall, this effect displays as a −15 mV shift in the inactivation curve in the presence of isoeugenol when compared to their controls, in the absence of this drug. This notion aligns with the classic idea of an inducible receptor for local anesthetics on VGSC that was previously described in studies on lidocaine’s modulatory effects on these channels [40,41]. These results are commonly associated with an increased affinity to the inactivated states of VGSC and it has been classically demonstrated to be part of the effects of lidocaine as a local anesthetic [42,43,44]. It is important to note that VGSC can become inactivated from closed states, which would explain the shift in the inactivation curves in the region of conditioning pulses’ membrane potentials when no recordable currents are detected [41].

We tested whether isoeugenol binds to inactivated states of VGSC with a higher affinity as compared to its binding to the channels’ resting states. To that end we used two approaches consisting of voltage clamp protocols that only contain membrane potential values that are associated with the resting state of the channels (−110 mV used as holding potential in this study) or with open/inactivated states of the channels (+20 mV used in this study). Membrane potentials between −110 mV and +20 mV would greatly increase the probability of the channels to populate pre-open closed states. Both approaches, in different ways, essentially test for the efficiency of the recovery from inactivation process in VGSC, and we used them in the absence (control) and in the presence of isoeugenol in order to learn if this drug affects the process.

First, we used the classic 3-pulse protocol that tests for the kinetics of the recovery from inactivation of the VGSC. When a drug specifically binds to the inactivated states of the VGSC, this recovering process is delayed as a function of the stabilization of the drug/inactivated-VGSC complex. Lidocaine is well known to greatly delay this process, and this drug commonly serves as a reference for new molecules in this regard. Isoeugenol delays this process of recovery from inactivation but the overall effect is not comparable to the one exerted by lidocaine as we recently replicated [26]. The weaker effect of isoeugenol on that recovering process is due to a stronger effect only on the fast component of the process which accounts for only 10–20% of the whole process (Figure 7).

Next, we used a time series of membrane depolarizations to +20 mV, from our common −110 mV holding potential, at 2 Hz and 5 Hz. This protocol tests for a cumulative decrease in the peak of I_Na_ as a proxy for insufficient recovery from inactivation. Once again, drugs like lidocaine greatly enhance this accumulated inhibition during the series. This cumulative deficit in the recovery from inactivation appears as a more intense blockade of the I_Na_ as the time series progresses. All other experiments shown here were performed at 0.2 Hz which was intended to avoid such accumulation between pulses of the respective time series. At 0.2 Hz, 50 ms pulses would be separated by 4950 ms, that is time enough for the full recovery from inactivation. At 2 Hz and 5 Hz, these interpulse periods are, respectively, 450 ms and 150 ms. Isoeugenol does not enhance I_Na_ inhibition during 2 Hz or 5 Hz time series of depolarization, in agreement with the recovery from inactivation kinetics approach described above.

We preliminarily conclude, therefore, that isoeugenol does not bind to inactivated states of VGSC with enhanced affinity compared to that of the resting states of the channels. These results are similar to what we observed with eugenol in our previous study. But how can a drug like isoeugenol shift the inactivation curve of VGSC without especially affecting the inactivated states of the channels? We asked ourselves the same question in a recent study comparing eugenol with lidocaine on their VGSC inhibition [26]. In that study, we argued, for the first time to our knowledge, that a shift in the inactivation curve does not necessarily indicate higher affinity of a drug to the inactivated state of VGSC. By evaluating the recovery from inactivation, a drug that binds to the inactive state with higher affinity would naturally delay this process. Similarly, by increasing the frequency of depolarizations, we increase the time the channels spend at a voltage that is associated with inactivation and the overall inhibition of VGSC would be expected to increase if a drug being tested interacts with the inactivated states with a higher affinity. Our data categorically demonstrate these are not cases for isoeugenol, despite this drug being capable of inducing a consistent and highly significant shift in the inactivation curves (Figure 6). More than that, we showed that at about a 50% overall blockade at the resting state with conditioning pre-pulses in the range of −140 mV (Figure 6C), the shift in the inactivation curves is in the order of 20 mV (Figure 6F).

Our suggestion to explain our data is that isoeugenol might interact with the pre-open closed states of VGSC to shift the inactivation curve of the channels to more negative potentials, as suggested in our previous studies [19,26]. Our data showing an isoeugenol-induced acceleration of the Na^+^ conductance activation, highly different from control, corroborates our hypothesis that isoeugenol binds to pre-open closed states of VGSC (Figure 3). In this notion the presence of the drug would bias the VGSC towards these states, making them quickly activatable when the membrane potential is depolarized to high voltages like +20 mV. It is important to note that this tentative conceptual hypothesis needs to be comprehensively tested in the future, and that it is beyond the scope of the present study. Simulations might help the understanding of these concepts. However, the addition to multiple pre-open closed states makes modeling unstable which requires imposing constraints to the method and manual adjustment in the kinetic parameters of the inter-state transitions of the channels. This approach will be used by us in the future to investigate this hypothesis.

We preliminarily propose that the direct interaction of isoeugenol with VGSCs is state-dependent as the inhibitory activity is enhanced by membrane depolarization. This effect is beneficial for pain management as the drug would act more effectively as neuronal activity is promoted by membrane depolarization. Nociceptive sensory neurons frequently exhibit ectopic firing and depolarized resting membrane potentials; these depolarizations increase the number of VGSCs in open or inactivated states. This mechanism phenomenologically parallels that of therapeutic drugs like lacosamide, which also preferentially target inactivated channels and show greater efficacy in neurons with chronically depolarized states. By targeting neurons in a hyperactive state while sparing normally polarized neurons, isoeugenol’s state-dependent inhibition may offer more robust anesthesia [45].

Our findings presented here suggest that isoeugenol acts like, but not the same as lidocaine as a modulator of VGSC. Lidocaine is an established and widely used local anesthetic in humans and animals. Clinically, lidocaine is used as a 2% solution that equates to an 85 mM solution. In vitro, the IC_50_ of the modulation of VGSC by lidocaine is around 1 mM, indicating isoeugenol is in the range of local anesthetics regarding affinity to bind to VGSC.

When we compare our data showing the inhibitory effects of isoeugenol, eugenol, and lidocaine on VGSC, we find that the two phenylpropenes act remarkably differently from lidocaine. Isoeugenol and eugenol, with different affinities, inhibit VGSC by supposedly binding to closed states, while lidocaine knowingly inhibits VGSC by binding to their hyperpolarized closed state and to the channels’ inactivated states. Interestingly, anethole, a phenylpropene like isoeugenol and eugenol, clearly interacts with the inactivated states of VGSC like lidocaine. We hypothesize that phenylpropenes possess various mechanisms of action that culminate with the inhibition of VGSC. Importantly, slight differences in the molecules of phenylpropenes, such as the ones between isoeugenol and anethole, can greatly change their inhibitory molecular mechanism on VGSC. These findings suggest that phenylpropenes are versatile molecules that can serve as archetypal molecules for the development of new drugs to block or to inhibit VGSC for benefits like analgesia, anti-seizure, antiarrhythmic, and others to humans and animals.

## 4. Materials and Methods

### 4.1. Cells Preparation

For our present study on the effects of isoeugenol on VGSC, we used cultured primary sensory neurons from rat dorsal root ganglia (DRG) as detailed below. Voltage-activated Na^+^ currents (I_Na_) from these DRG neurons were isolated and recorded under voltage-clamp for our tests with isoeugenol.

All animals were handled in compliance with the Guide for the Care and Use of Laboratory Animals by the U.S. National Institutes of Health (Guide for the Care and Use of Laboratory Animals, 8th edition, 2011; https://www.ncbi.nlm.nih.gov/books/NBK54050/, accessed on 8 June 2025) [46]. DRGs from 1 to 3 day-old rat lumbar sections were dissected and split into two or three pieces while in ice cold Dulbecco’s Modified Eagle’s Medium (DMEM, Millipore Sigma, St. Louis, MO, USA). These split ganglia are easily digested with a single 15 min period in a 0.25% trypsin in a Ca^2+^- and Mg^2+^-free Early’s balanced salt solution (EBSS) containing (mM) 132.8 NaCl, 5.3 KCl, 1 NaH_2_PO_4_, 5.5 glucose and 10 HEPES, pH 7.4. After 15–20 min digestion, softened DRGs were further reduced with fire-polished Pasteur pipettes in a Ca^2+^, Mg^2+^-free EBSS containing 5 U/mL DNAse (type I, Sigma, St. Louis, MO, USA), 0.15% of trypsin inhibitor (type IS, Sigma) and supplemented with 10% fetal calf serum. All reagents were purchased from Sigma, St. Louis, MO, USA unless otherwise noted. After pelleting by mild centrifugation, monodispersed cells were resuspended in Dulbecco’s Modified Eagle’s Medium (DMEM, Sigma) supplemented with 10% fetal calf serum, 100 UI/mL penicillin, 100 μg/mL streptomycin, and seeded on glass coverslips treated with poly-L-lysine. Cell cultures were kept at 37 °C in a 5% CO_2_ atmosphere until just before experiments. Neurons remained viable for electrophysiology experiments for up to 7 days after the establishment of the cell culture.

### 4.2. Isoeugenol Solutions

Isoeugenol (Millipore Sigma, St. Louis, MO, USA, CAS Number: 97-54-1, Mol. Weight 164.20 g/mol) was prepared as a 1 M stock solution in ethanol and stored at −20 °C. Right before an experiment, this 1 M solution was diluted in the bath solution (see recipe below) for a given concentration of isoeugenol (0.001–30 mM) and sonicated. The maximal final concentration of ethanol used in this study was 0.73% vol/vol, which, according to our own data [26] and data from others [47,48], inhibits I_Na_ in approximately 10%. Importantly, this inhibition does not affect the voltage-dependence or the kinetics of the I_Na_. During the recordings, the solution containing isoeugenol at a given concentration was applied directly to the cell under experimentation to minimize biases in the real concentration to which the cell is exposed, and to avoid delays in the I_Na_ inhibition. All other chemical and reagents used in this study were at least of reagent grade and purchased from Millipore Sigma, St. Louis, MO, USA.

### 4.3. Electrophysiology

I_Na_ were recorded using voltage-clamping with the conventional whole-cell patch-clamp configuration. Patch pipettes were fabricated from borosilicate glass capillaries using a model P-97 micropipette puller (Sutter Instrument, Novato, CA, USA). Patch pipettes were pulled to achieve initial resistances averaging 2 MΩ when filled with an intracellular solution (see recipe below) and dipped into bathing solution. Intracellular solution consisted of (mM) 10 NaCl, 150 CsF, 10 TEA-chloride, 1 ATP, 4.5 MgCl_2_, 9 EGTA, 10 HEPES, pH 7.3. CsCl and TEA-chloride were used to eliminate K^+^ currents. Bathing solution consisted of (mM) 82 choline chloride, 50 NaCl, 1.2 MgCl_2_, 1.8 CaCl_2_, 1 CoCl_2_, 4 KCl, 5 glucose, 10 HEPES, pH 7.4. CoCl_2_ was used to eliminate voltage activated Ca^2+^ currents from the recordings. All reagents were purchased from Sigma, St. Louis, MO, USA unless otherwise noted. Typical access resistance values were below 3 MΩ. When the giga-seal was achieved for the whole-cell patch clamp experiments. The patch clamp amplifier, an Axopatch 200B patch-clamp amplifier (Molecular Devices, Sunnyvale, CA, USA), as usual, was driven by a waveform generated by software Clampex 10 (Molecular Devices, Foster City, CA, USA) and made analog by a Digital-to-Analog (DA) converter (model 1322, Molecular Devices, Foster City, CA, USA). The current recordings were low-pass filtered at 2 kHz by a built-in Bessel filter in the amplifier, sampled at 25–50 kHz in 16-bits digital levels by an Analog-to-Digital (AD) converter (model 1322, Molecular Devices, Foster City, CA, USA) and recorded on a hard disk for analysis. The cell membrane capacitance was canceled, and access resistance was routinely compensated (85% for both prediction and compensation; lag set to 10 μs). P/-4 protocol [49] was used to eliminate uncompensated capacitive currents and leak currents from the recorded data. However, this technique was not used when testing for the potential use-dependent block by isoeugenol to avoid underestimation in the overall recovery from inactivation processes. We used a −110 mV holding potential as a standard procedure to always remove channels from inactivation when not pulsing. In addition, when possible, we pulsed at 0.2 Hz throughout the time series of the protocol for the same reason. All recordings were performed between 20 and 23 °C. The recording chamber was continuously perfused with bath solution to avoid unstirred layers and maintained between 20 and 23 °C

### 4.4. Data Analysis and Graphing

Scientific data was processed, fitted with equations, analyzed, and plotted using Clampfit 11.4 (Molecular Devices, Foster City, CA, USA), GraphPad Prism (GraphPad 10.5 Software, LLC, La Jolla, CA, USA), Origin 8 (OriginLab, Northampton, MA, USA), and Microsoft Excel (Microsoft, Redmond, WA, USA). Plotted data as graphs represent mean values and the vertical bars indicate the standard error of the mean (SEM). Our experiments were conducted with repeated measures (paired data) to highlight the effects of isoeugenol despite the different I_Na_ expressed in different neurons utilized in this study.

### 4.5. Fitting Models

Dose response curves were fitted with the Hill’s formalism:

(1) $$Fractional\;I_{Na}=\frac{{\left[ISO\right]}^{nH}}{{\left[ISO\right]}^{nH}+{{IC}_{50}}^{nH}}$$

where the Fractional I_Na_ is the remaining I_Na_ after inhibition by isoeugenol, IC_50_ is the concentration of isoeugenol that inhibits 50% of the I_Na_ and nH is the Hill’s coefficient.

I_Na_ values from the current–voltage (I–V) curves were transformed into Na^+^-conductances–voltage (G–V) curves by using Ohm’s law:

(2) $$G_{Na}=\frac{I_{Na}}{V_{m}-V_{r}}$$

where G_Na_ is the Na^+^ conductance, I_Na_ is the Na^+^ current, V_m_ is the membrane potential and V_r_ is the reversal potential of I_Na_.

Na^+^ conductance was fitted by the Hodgkin and Huxley model as follows:

(3) $$G_{Na}=G_{max}\times {\left(1-e^{-t/Tau\;m}\right)}^{3}\times e^{-t/Tau\;h}$$

where G_Na_ is the instantaneous Na^+^ conductance, G_max_ is the maximal Na^+^ conductance, *t* is the time, Tau m is the time constant of activation, and Tau h is the time constant of inactivation.

Na^+^ conductance activation by voltage (G–V) curves were fitted by the following equation:

(4) $$Normalized\;{Na}^{+}conductance=\frac{1}{1+e^{\left(\frac{V_{0.5-act}-V_{m}}{Voltage\;sensitivity}\right)}}$$

where Normalized Na^+^ conductance is the fractional conductance activated at a given membrane potential V_m_. This parameter is the absolute Na^+^ conductance in non-normalized plots. V_0.5-act_ is the membrane potential for half-maximal Na^+^ conductance activation (the midpoint) and voltage sensitivity is the minimal membrane potential change that is associated with increase or decrease in the Na^+^ conductance by e-fold, also known as the maximal slope of the curve.

Na^+^ currents inactivation by voltage curves (inactivation curves) were fitted by the following equation:

(5) $$Fractional\;I_{Na}=1-\frac{1}{1+e^{\left(\frac{V_{0.5-inact}-V_{cp}}{Voltage\;sensitivity}\right)}}$$

where Fractional I_Na_ is the Na^+^ current after the conditioning pre-pulse (V_cp_) voltage period, V_0.5−iNact_ is the V_cp_ that inactivates half of I_Na_, and the is the minimal membrane potential change that is associated with inactivation of I_Na_ by e-fold, also known as the maximal slope of the curve.

The I_Na_ recovery from inactivation process was fitted with a double exponential as follows:

(6) $$I_{Na}=\%\;Fast\;comp\times \left(1-e^{-t/Fast\;Tau}\right)+\%\;Slow\;comp\times \left(1-e^{-t/Slow\;Tau}\right)$$

where I_Na_ is the recovered I_Na_ associated with a recovery period t, Fast Tau is the time constant of the faster recovery from inactivation process, % Fast comp is the fractional % of the fast component, Slow Tau is the time constant of the slower recovery from inactivation process, % Slow comp is the fractional % of the slow component (equivalent to 100 -% Fast comp).

### 4.6. Statistical Analysis

Data from individual cells were treated individually, including for fitting analyses. Pooled fitting parameters from different groups, e.g., control vs. isoeugenol (its presence) were compared using Paired *t* test to detect consistent changes in the parameters that relates to the drugs. Levels of significance were * *p* < 0.05, ** *p* < 0.01, *** *p* < 0.001 and **** *p* < 0.0001. The whole curves were compared using Two-way ANOVA.

## 5. Conclusions

We conclude, based on our data, that isoeugenol has significant and fully reversible modulatory effects on VGSC, inducing dose-dependent inhibition of I_Na_ with a suggestive state-dependent additional biding effect. The drug is safe to use, and it is effective in a reasonable range of concentrations for currently used therapeutic drugs. The putative effect on pre-open closed states might be an interesting area to explore in the future since membrane depolarizations in the order of membrane potential fluctuations associated with pathologies could trigger a more intense VGSC inhibition by eugenol. However, further studies must be conducted to understand the interaction of isoeugenol on the pre-open closed states of the channels, and its similarities to the characteristics of lidocaine. The present study contributes to the field of natural products and new drugs. Our study paves the way for possible future studies to establish isoeugenol or new derivatives as novel drugs to be used in humans and animals as anesthetic to treat pain or other excitable tissues disturbances.

## Figures and Tables

**Table 1:** Fit parameters from analyses of I_Na_ voltage dependent activation and inactivation processes.

	Activation (n = 7)		Inactivation (n = 6)	
	Control	Isoeugenol 1 mM	Control	Isoeugenol 1 mM
V_r_ shift (mV)	44.4 ± 3.18	42.3 ± 3.53 ^ns^	N/A	N/A
G_max_ (nS)	171.7 ± 24.88	119.0 ± 25.09 *	180.9 ± 21.88	100.75 ± 22.98 *
V_0.5_ (mV)	−14.2 ± 2.76	−10.7 ± 3.72 ^ns^	−42.3 ± 2.95	−57.3 ± 2.11 **
Paired delta V_0.5_ (mV)	3.56 ± 1.86		−15.1 ± 3.37 **	
Voltage sensitivity (mV/e-fold)	4.0 ± 0.50	5.34 ± 0.36 ^ns^	9.4 ± 0.26	10.9 ± 0.42 ^ns^

Key (Paired *t* test): ns, not significant; * *p* < 0.05; ** *p* < 0.01. N/A, not applicable.

**Table 2:** Fit parameters from analysis of I_Na_ time dependent recovery from inactivation.

	Recovery from Inactivation (n = 5)	
	Control	Isoeugenol 1 mM
% fast component	84.3 ± 3.15	87.8 ± 2.60 ^ns^
Fast component tau (ms)	8.5 ± 0.70	13.0 ± 0.98 **
Slow component tau (ms)	75.9 ± 11.40	181.6 ± 30.51 *

Key (Paired *t* test): ns, not significant; * *p* < 0.05; ** *p* < 0.01.

## Data Availability

The data presented in this study are available from the corresponding author upon reasonable request.
